# Endoplasmic reticulum stress in bone marrow-derived cells prevents acute cardiac inflammation and injury in response to angiotensin II

**DOI:** 10.1038/cddis.2016.164

**Published:** 2016-06-09

**Authors:** T-T Li, L-X Jia, W-M Zhang, X-Y Li, J Zhang, Y-L Li, H-H Li, Y-F Qi, J Du

**Affiliations:** 1 Beijing Anzhen Hospital, Capital Medical University, Beijing 100029, China; 2 The Key Laboratory of Remodeling-Related Cardiovascular Diseases, Ministry of Education, Beijing 100029, China; 3 Beijing Collaborative Innovation Center for Cardiovascular Disorders, Beijing 100029, China; 4 Beijing Institute of Heart, Lung & Blood Vessel Disease, Beijing 100029, China

## Abstract

Inflammation plays an important role in hypertensive cardiac injury. The endoplasmic reticulum (ER) stress pathway is involved in the inflammatory response. However, the role of ER stress in elevated angiotensin II (Ang II)-induced cardiac injury remains unclear. In this study, we investigated the role of ER stress in Ang II-induced hypertensive cardiac injury. Transcriptome analysis and quantitative real-time PCR showed that Ang II infusion in mice increased ER stress-related genes expression in the heart. C/EBP homologous protein (CHOP) deficiency, a key mediator of ER stress, increased infiltration of inflammatory cells, especially neutrophils, the production of inflammatory cytokines, chemokines in Ang II-infused mouse hearts. CHOP deficiency increased Ang II-induced cardiac fibrotic injury: (1) Masson trichrome staining showed increased fibrotic areas, (2) immunohistochemistry staining showed increased expression of *α*-smooth muscle actin, transforming growth factor *β*1 and (3) quantitative real-time PCR showed increased expression of collagen in CHOP-deficient mouse heart. Bone marrow transplantation experiments indicated that CHOP deficiency in bone marrow cells was responsible for Ang II-induced cardiac fibrotic injury. Moreover, TUNEL staining and flow cytometry revealed that CHOP deficiency decreased neutrophil apoptosis in response to Ang II. Taken together, our study demonstrated that hypertension induced ER stress after Ang II infusion. ER stress in bone marrow-derived cells protected acute cardiac inflammation and injury in response to Ang II.

Hypertension induces damage to heart, and inflammation plays an important role.^1, 2, 3^ As a central effector of hypertension, dysregulated angiotensin II (Ang II) triggers a set of inflammatory responses, leading to organ damage.^4, 5^ In cardiac injury, Ang II causes endothelial cell injury and platelet activation and increases local production of cytokines, chemokines and infiltration of inflammatory cells.^6^ We and others have demonstrated that the bone marrow (BM)-derived inflammatory cells, including macrophages, T cells and neutrophils, all played critical roles in Ang II-induced cardiac injury.^4, 7, 8, 9^ However, how the inflammatory pathways were activated and how they were regulated remains to be fully investigated.

The endoplasmic reticulum (ER) is the site of synthesis and maturation of proteins designed for secretion or for localization on the cell membrane. Various types of stress from both inside and outside cells disturb ER function, thus causing unfolded or misfolded proteins to accumulate in the ER.^10^ To improve and maintain the ER functions against such stresses, the ER stress response pathway is activated. All three upstream signaling pathways that mediate ER stress, including the inositol requiring kinase 1*α*/X-box-binding protein 1, activating transcription factor (ATF)6, and protein kinase-like ER kinase/eukaryotic translation initiation factor 2/ATF4 pathways, intersect at the transcription factor C/EBP homologous protein (CHOP) to initiate apoptosis and inflammation.^11, 12^ Previous studies have demonstrated that ER stress was involved in various diseases, including cardiovascular diseases,^10^ obesity,^13^ acute kidney injury (AKI)^14^ and so on. But the role of ER stress in different diseases was different. For example, CHOP deficiency alleviated myocardial reperfusion injury by inhibiting myocardial apoptosis and inflammation,^15^ while ablation of CHOP increased the acute phase mortality in mice with myocardial infarction.^16^ CHOP-deficient mice were reported to be resistant to lung inflammation and injury induced by lipopolysaccharide (LPS) infusion,^12^ whereas CHOP deficiency resulted in elevated LPS-induced inflammation and kidney injury.^14^ However, whether CHOP-mediated ER stress is involved in Ang II-induced cardiac injury is unknown and the role of ER stress in this process remains unclear.

It is known that inflammatory response plays a critical role in Ang II-induced cardiac injury. CHOP-mediated ER stress was involved in various diseases by regulating apoptosis and inflammation. So we hypothesized that ER stress is involved in regulating apoptosis/survival of inflammatory cells during cardiac inflammation and injury in response to elevated Ang II. In the present study, we aimed to investigate whether CHOP-mediated ER stress could be activated in Ang II-induced acute cardiac injury and explore the role of ER stress in this process. We found that hypertension induced ER stress after Ang II infusion. ER stress in BM-derived cells protected acute cardiac inflammation and injury in response to Ang II.

## Results

### Ang II infusion induced ER stress in heart

**Figure 1:**
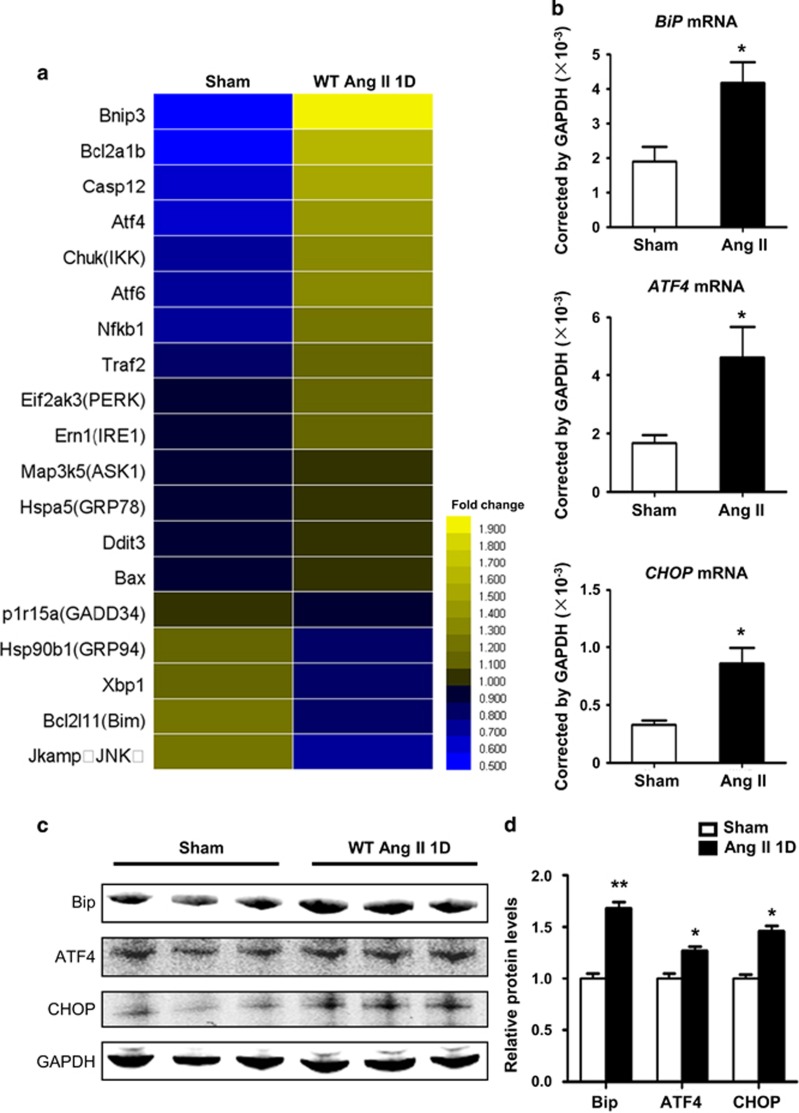
Hypertension induced ER stress after Ang II infusion. (**a**) RNA Seq was performed in Ang II-infused WT mouse hearts at day 1 and ER stress-related genes were analyzed. (**b**) *Grp78/BiP, ATF4* and *CHOP* mRNA levels in Ang II-infused WT mouse hearts at day 1 were determined by qRT-PCR. Values were normalized to GAPDH (*n*=6 in each group). (**c**) BiP, ATF4 and CHOP protein levels in Ang II-infused WT mouse hearts at day 1 were determined by western blot. (**d**) Bar graph shows the quantifications of BiP, ATF4 and CHOP relative to GAPDH (*n*=3 in each group). *P<0.05 compared with the Sham group

To investigate whether ER stress is activated in Ang II-induced cardiac inflammation and injury, we performed whole-genome RNA sequencing of sham and Ang II-infused mouse hearts at day 1. We found genes associated with ER stress were mostly upregulated in the heart (Figure 1a). The mRNA expressions of *Grp78/BiP, ATF4, CHOP, GRP94, GADD34* and *Xbp1* in sham or Ang II-infused hearts were confirmed by quantitative real-time PCR (Figure 1b and Supplementary Figure SIA). The protein levels of BiP, ATF4 and CHOP were also upregulated at day 1 after Ang II infusion compared with sham. To clarify whether ER stress can be activated at day 3 and day 7 after Ang II infusion, we detected the RNA expression level of Bip, ATF4 and CHOP at different time points. The results showed the expression of Bip,ATF4 or CHOP gradually was initially increased at day 1 and decreased at day 3 and 7 after Ang II infusion (Supplementary Figure SIB). These results demonstrated hypertension induced ER stress after Ang II infusion.

### Knockout of CHOP, a key mediator of ER stress, increased cardiac inflammation in response to Ang II

**Figure 2:**
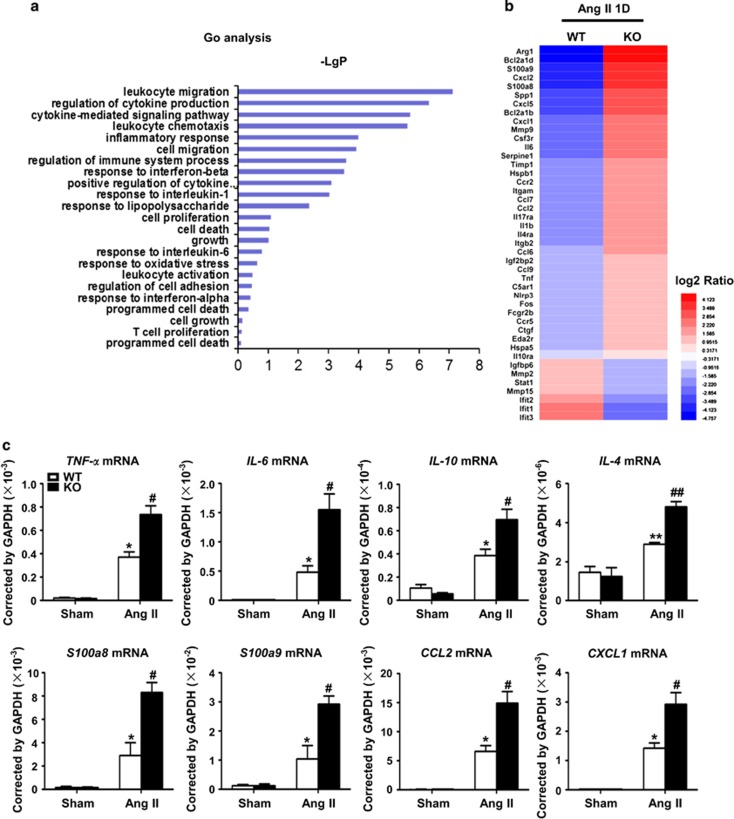
CHOP deficiency increased Ang II-induced expression of inflammatory factors. (**a**) RNA Seq was performed in Ang II-infused WT and CHOP KO mouse hearts at day 1. Gene Ontology (GO) analysis of log10-transformed *P*-values from biological process of GO terms. (**b**) Upregulated genes were shown as heat map. (**c**) qRT-PCR validation for RNA-seq data. The mRNA levels of *TNF-α, IL-6, IL-10, IL-4 S100a8/a9, CCL2* and *CXCL1* in Ang II-infused WT and CHOP KO hearts at day 1. *n*=6 per group. *P<0.05 compared with the Sham group. ^#^*P*<0.05 compared with the WT group. ^##^*P*<0.01 compared with the WT group

**Figure 3:**
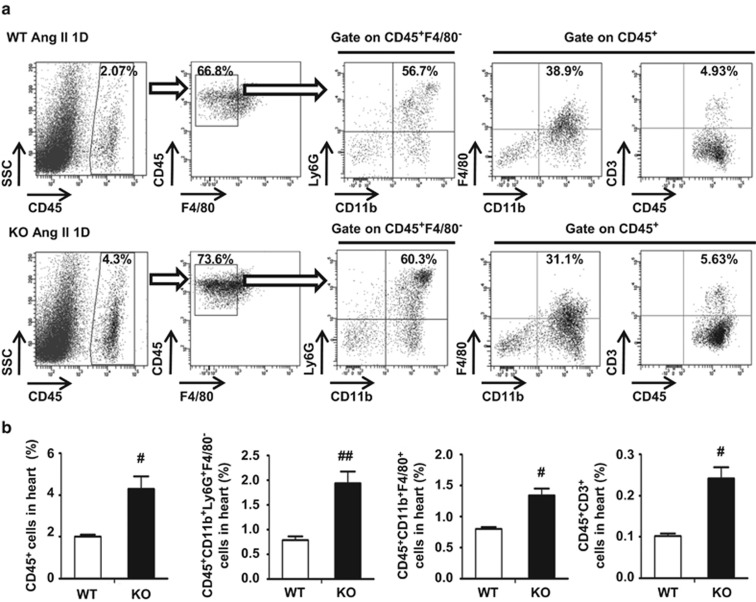
CHOP deficiency increased Ang II-induced infiltration of inflammatory cells in the heart. (**a**) Flow cytometry analysis of CD45^+^ leukocytes, CD45^+^CD11b^+^Ly6G^+^F4/80^−^neutrophils, CD45^+^CD11b^+^F4/80^+^macrophages or CD45^+^CD3^+^ T cells were performed in Ang II-infused WT and CHOP KOhearts at day 1 (*n*=4 in each group). (**b**) Bar graph shows the percentage of cells in the heart (*n*=4 in each group). ^#^*P*<0.05 compared with the WT group. ^##^*P*<0.01 compared with the WT group

Three different pathways which induce ER stress, all intersect at CHOP to initiate apoptosis and inflammation.^17^ Therefore, CHOP KO mice were used to investigate the role of ER stress in Ang II-induced cardiac injury. RNA sequencing showed that CHOP deficiency significantly increased the expression of inflammatory genes, including cytokine and chemokine secretion at day 1 after Ang II infusion (Figures 2a and b). The increases in mRNA levels of cytokines and chemokines, including *TNF-α, IL-6, IL-10, IL-4 S100a8, S100a9*, chemokine (C-X-C motif) ligand 1 (*CXCL1*) or chemokine (C-C motif) ligand 2 (*CCL2*), were confirmed by RT-PCR (Figure 2c). Accordingly, inflammatory cell infiltration into the heart was examined by flow cytometry. As shown in Supplementary Figure SII and Figure 3, Ang II infusion significantly increased the infiltration of CD45^+^ leukocytes, CD45^+^CD11b^+^Ly6G^+^F4/80^−^ neutrophils, CD45^+^CD11b^+^F4/80^+^ macrophages and CD45^+^CD3^+^T cells into the heart at day 1 after Ang II infusion, while CHOP deficiency increased the inflammatory response.

To document changes in the spleen of these mice after Ang II infusion, inflammatory cell infiltration into the spleen was examined by flow cytometry. As is shown in Supplementary Figure SIII, the percentage of CD45^+^CD11b^+^Ly6C^+^ monocytes, CD45^+^CD11b^+^F4/80^+^ macrophages or CD45^+^CD11b^+^Ly6G^+^F4/80^−^ neutrophils in the spleen was decreased at day 1 after Ang II infusion in wild-type (WT) mice, while CHOP deficiency did not affect this inflammatory response.

### CHOP deficiency increased Ang II-induced cardiac fibrotic injury

**Figure 4:**
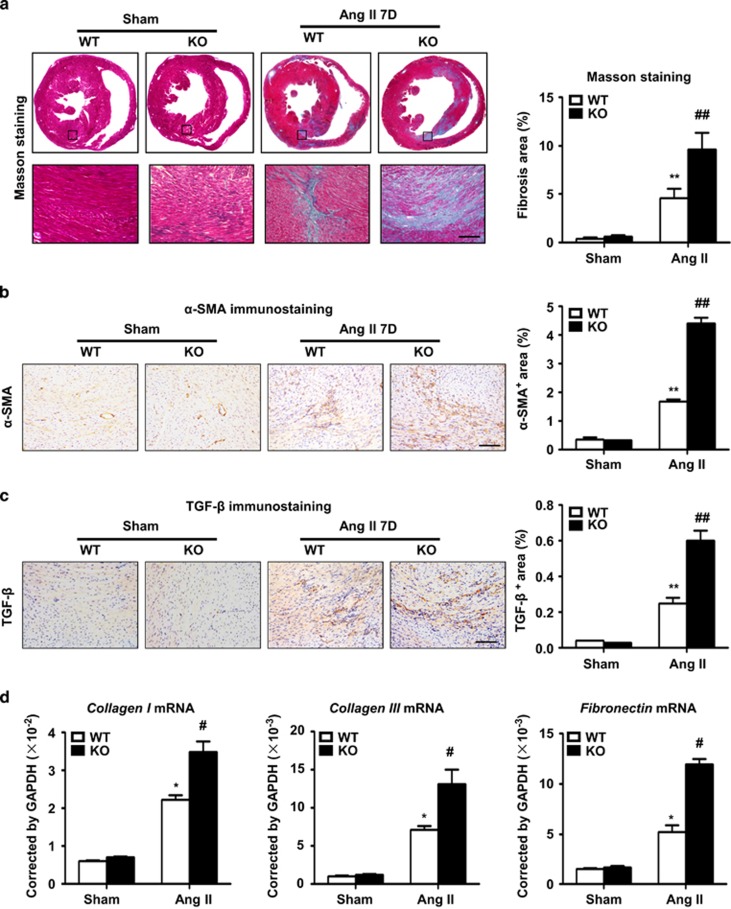
CHOP deficiency increased Ang II-induced cardiac injury. (**a**) Masson staining of fibrosis and area quantitation in WT and CHOP KO mouse hearts at day 7 in the sham or Ang II infusion group (scale bars, 100 *μ*m; *n*=6 in each group). The representative pictures and quantification of (**b**) *α*-SMA and (**c**) TGF-*β* staining in WT and CHOP KO mouse hearts at day 7 in the sham or Ang II infusion group (scale bars, 100 *μ*m; *n*=6 in each group). (**d**) *Collagen I, collagen III* and *fibronectin* mRNA levels in WT and CHOP KO mouse hearts at day 7 in the sham or Ang II infusion group (*n*=6 in each group). *P<0.05 compared with the Sham group. **P<0.01 compared with the Sham group. ^#^*P*<0.05 compared with the WT group. ^##^*P*<0.01 compared with the WT group

Given that CHOP deficiency aggravates Ang II-induced cardiac inflammation, we assessed whether this event leads to severe cardiac injury and remodeling. Ang II-induced cardiac injury is characterized by the expression of profibrotic and extracellular matrix genes, including transforming growth factor-*β* (*TGF-β*), *α*-smooth muscle actin (*α-SMA*) and collagen.^18^ CHOP deficiency markedly increased extracellular matrix deposition, determined by Masson's trichrome staining (Figure 4a). There were markedly more positive areas of TGF-*β* and *α*-SMA staining in Ang II-treated CHOP knockout hearts (Figures 4b and c). These results were further confirmed at the gene expression level, as Ang II infusion induced the mRNA levels of *collagen I, collagen III* and *fibronectin* in CHOP knockout hearts were significantly increased at day 7 compared with WT mice (Figure 4d). However, CHOP deficiency did not affect blood pressure, cardiac hypertrophy or cardiac function of Ang II-treated mice (Supplementary Figures SIVA, B and C). Thus, our results demonstrated that CHOP deficiency increased Ang II-induced cardiac fibrotic injury and remodeling.

### CHOP deficiency in BM-derived cells was responsible for Ang II-induced cardiac injury

**Figure 5:**
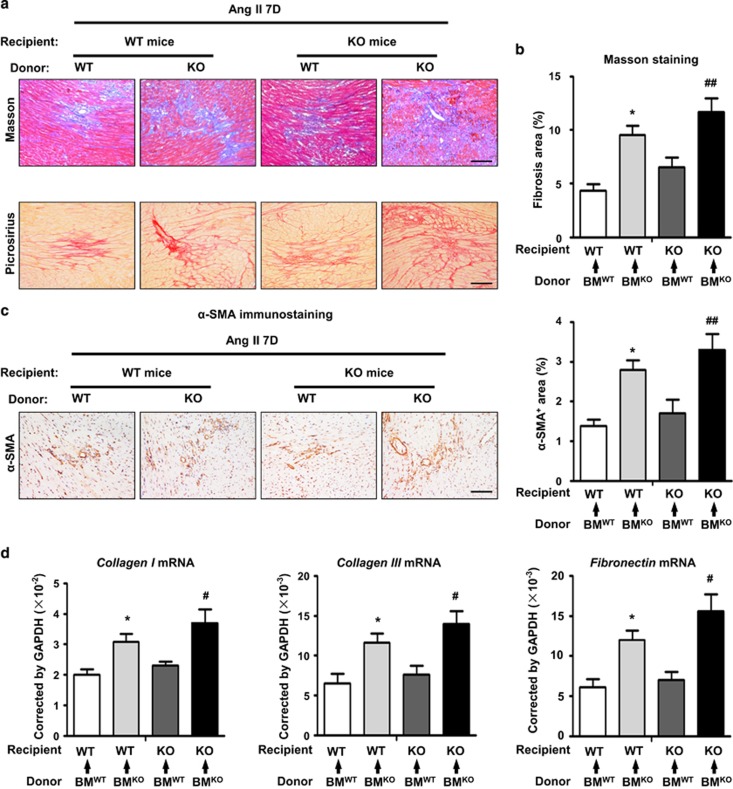
CHOP deficiency in bone marrow-derived cells promoted Ang II-induced cardiac injury. (**a**) Masson's trichrome and picrosirius red staining in Ang II-infused heart tissues from bone marrow chimeric mice at day 7 (scale bars, 100 *μ*m). (**b**) Quantification of fibrotic areas in heart sections with Masson's trichrome staining (*n*=4 in each group). (**c**) The representative pictures and quantification of *α*-SMA in staining in Ang II-infused heart tissues from bone marrow chimeric mice at day 7 (scale bars, 100 *μ*m; *n*=4 in each group). (**d**) *Collagen I, collagen III* and *fibronectin* mRNA levels in in Ang II-infused heart tissues from bone marrow chimeric mice at day 7 (*n*=4 in each group). *P<0.05 compared with BM^WT^ to WT. ^#^*P*<0.05 compared with BM^WT^ to KO. ^##^*P*<0.01 compared with BM^WT^ to KO

We performed BM transplantation experiments to address whether CHOP deficiency in heart cells or BM-derived cells is responsible for aggravated Ang II-induced cardiac injury. BM chimeric mice were reconstituted after transplantation of WT or CHOP-deficient BM cells. Two months after BM transplantation, mice underwent Ang II infusion. After 7 days of Ang II infusion, mice that received CHOP knockout BM showed a severe cardiac injury compared with those that received WT BM, regardless of the genotype of the recipient mice, as demonstrated by Masson's trichrome staining, picrosirius red staining, *α*-SMA staining and qRT-PCR analysis of fibrogenic genes (*collagen I, collagen III* and *fibronectin*) (Figures 5a–d). Thus, CHOP expression in BM cells rather than cardiac cells is critical for cardiac injury.

### CHOP deficiency decreased the apoptosis of neutrophils in hearts

**Figure 6:**
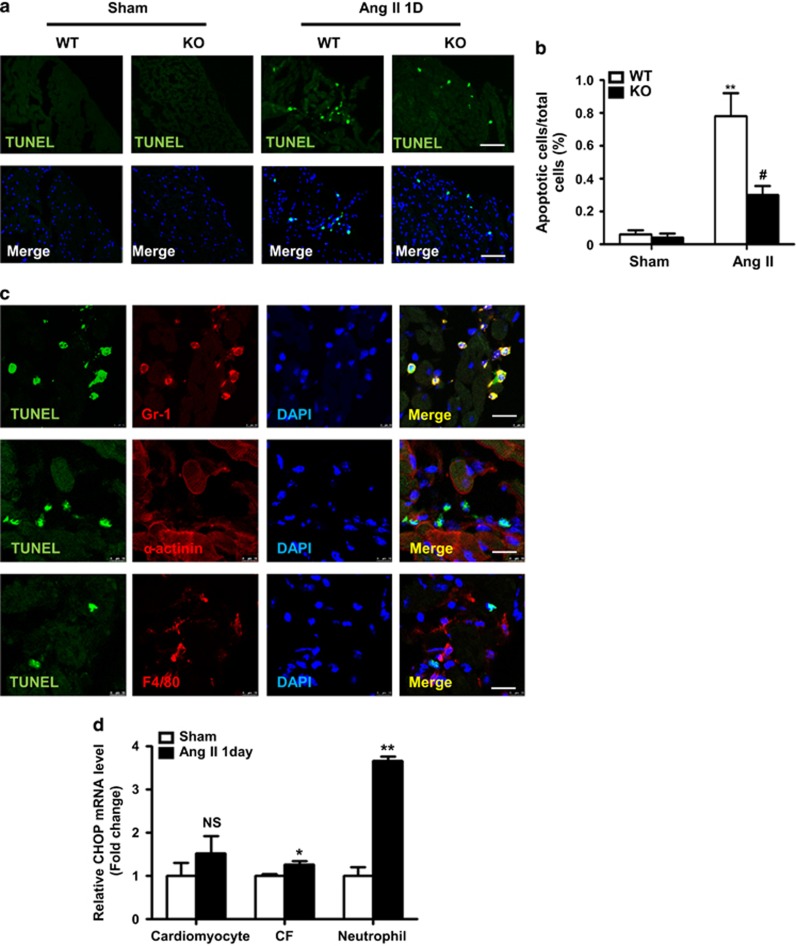
CHOP deficiency decreased neutrophil apoptosis. (**a**) TUNEL staining in Ang II-infused WT and CHOP KO mouse hearts at day 1 and (**b**) the quantification of TUNEL-positive cell number per high-power field (scale bars, 50 *μ*m; *n*=4 in each group). (**c**) Immunofluorescence staining stained for TUNEL (green) and Gr1 (red), F4/80 (red), *α*-actinin (red) in Ang II-infused WT mouse hearts at day 1. Nuclei were shown in blue with 4′,6-diamidino-2-phenylindole (DAPI) staining (scale bars, 10 *μ*m). (**d**) Graphic presentation shows mRNA expression level of CHOP in different cell populations (cardiomyocyte, CF, neutrophil) that were sorted from WT mice at day 1 after Ang II infusion or sham by flow cytometry (*n*=3 in each group). *P<0.05 compared with the Sham group.**P<0.01 compared with the Sham group. ^#^*P*<0.05 compared with the WT group. NS indicates not significant, compared with the Sham group

It is known that ER stress also results in apoptosis.^19^ We next performed TUNEL staining on heart sections of WT and CHOP KO mice at day 1 after Ang II infusion. As is shown in Figure 6a, TUNEL staining revealed that CHOP deficiency significantly decreased cell apoptosis in Ang II-infused hearts compared with that in WT mice. Co-staining of TUNEL and Gr1 (neutrophils), *α*-actinin (cardiomyocytes) and F4/80 (macrophages) showed that the apoptotic cells were mainly neutrophils, but not macrophages or cardiomyocytes (Figures 6a–c). Furthermore, we isolated cardiomyocytes, cardiac fibroblasts (CFs) and neutrophils at day 1 after Ang II infusion. And we detected the CHOP expression in these cells (Figure 6d). The results showed the fold change of CHOP mRNA expression was highest in CD45^+^CD11b^+^Ly6G^+^ neutrophils compared with CD45^−^PDGFR*α*^−^CD31^−^ cardiomyocytes and CD45^−^PDGFR*α*^+^CD31^−^ CFs. This results indicated that neutrophil was prone to ER Stress and apoptosis compared with cardiomyocyte and CF.

### ER stress was also involved in neutrophil apoptosis *in vitro*

**Figure 7:**
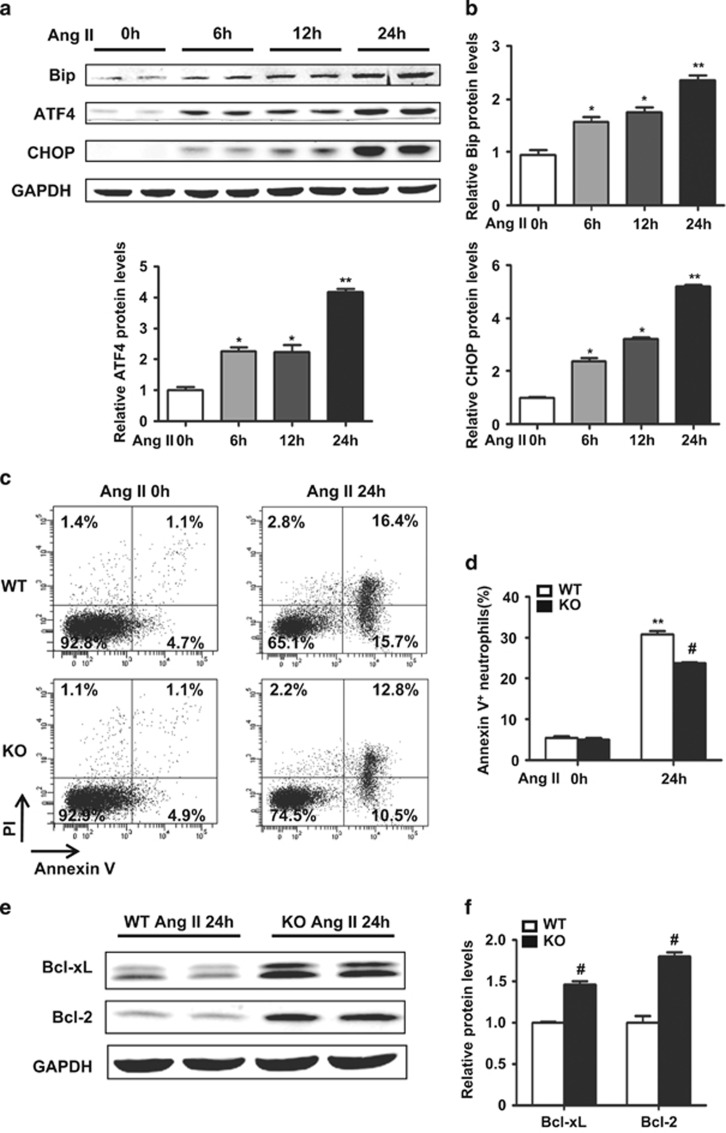
ER stress was involved in neutrophil apoptosis *in vitr*o. (**a**) BiP, ATF4 and CHOP protein levels in mouse neutrophils stimulated with Ang II for the indicated times. (**b**) Bar graph shows the quantifications of BiP, ATF4 and CHOP relative to GAPDH (*n*=4 in each group). (**c**) The apoptosis of WT and CHOP KO mouse neutrophils was analyzed by annexin V and PI staining stimulated with Ang II (1 *μ*mol/l) for 24 h. (**d**) Neutrophil apoptosis was calculated as the percentage of annexin V^+^ cells (both annexin V^+^PI^−^ and annexin V^+^PI^+^, *n*=3 in each group) to total cells. (**e**) Western blot analysis of Bcl-XL and Bcl-2 in WT and CHOP KO mouse neutrophils stimulated with Ang II (1 *μ*mol/l) for 24 h *in vitro*. (**e**) Bar graph shows the quantifications of Bcl-XL and Bcl-2 in relative to GAPDH (*n*=4 in each group). *P<0.05 compared with time zero. **P<0.01 compared with time zero. ^#^*P*<0.05 compared with the WT group

We then examined whether ER stress was involved in neutrophil apoptosis *in vitro.* Neutrophils were sorted by flow cytometry from murine BM and cultured with Ang II (1 *μ*mol/l) for different time. Western blot analysis showed the protein levels for BiP, ATF4 and CHOP were significantly upregulated in a time-dependent manner in neutrophils treated with Ang II (Figures 7a and b). Flow cytometry analysis of neutrophil apoptosis *in vitro* showed that CHOP deficiency prolonged neutrophil survival, comparing with WT neutrophil (Figures 7c and d). The antiapoptotic protein levels for Bcl-XL and Bcl-2 were both higher in CHOP-deficient neutrophils than those in WT neutrophils (Figures 7e and f).

## Discussion

In the present study, we investigated the role of ER stress in Ang II-induced cardiac inflammation and injury. Our results showed that Ang II infusion in mice increased ER stress-related proteins expression in the heart. Deficiency of CHOP, a key mediator of ER stress, increased Ang II-induced cardiac inflammation and injury. CHOP deficiency in BM-derived cells was responsible for Ang II-induced cardiac injury. Taken together, our study demonstrated that ER stress in BM-derived cells protects acute cardiac inflammation and injury in response to Ang II.

Ang II-induced cardiac injury, manifesting as cardiac fibrosis, has long been attributed to inflammatory responses.^4^ In this process, Ang II triggered a series of inflammatory responses acting by causing both hemodynamic and nonhemodynamic effects.^20^ Neutrophils are the most abundant inflammatory cells at the early stages of injury. When neutrophils degranulate and die, macrophages are recruited. During this initial leukocyte migration phase, the activated neutrophils and macrophages produce cytokines and chemokines, which amplify the inflammatory response.^1^ Subsequently, macrophages and T cells become activated and secrete profibrotic cytokines such as TGF-*β*, which in turn further activate the fibroblasts.^20^ Then activated fibroblasts transform into *α*-SMA-expressing myofibroblasts. An increased number of myofibroblasts, the dominant source of extracellular matrix production, results in deposition of collagen and cardiac injury.^20^ We and others have demonstrated that BM-derived cells, including macrophages, T cells and neutrophils, worked together to regulate the inflammatory environment and fibrosis.^4, 7, 8, 9, 21, 22, 23^ ER stress can be induced by multiple stimuli and it was reported to be involved in various diseases, including cardiovascular diseases,^10^ obesity,^13^ ^AKI14^ and so on. Although the role of ER stress in different diseases is different, we demonstrated that ER stress in BM-derived cells protected acute cardiac inflammation and injury in response to Ang II. Data from the current study revealed that the cardiac inflammatory response was increased in CHOP-deficient mice. We found that CHOP deficiency increased the infiltration of neutrophils, macrophages and T cells into the heart. Among these inflammatory cells, neutrophils increased mostly in CHOP-deficient mice compared with WT mice. The hearts of Ang II-treated CHOP-deficient mice exhibited a more significant increase in chemokine *S100a8* and *S100a9* mRNA levels, which can be produced by neutrophils. CHOP deficiency decreased the apoptosis of neutrophils in hearts. Neutrophils play an important role in acute injury.^24, 25^ They are the first responders of inflammatory cells to migrate towards the site of inflammation during the acute phase of inflammation.^26, 27, 28^ Neutrophils have a rapid rate of turnover, mainly through apoptosis and removal by phagocytosis *in situ*,^29^ which is a hallmark of inflammation resolution.^30^ Neutrophil apoptosis prevents release of cytotoxic neutrophil contents, averting unnecessary host tissue damage and inflammation.^31, 32^ Deficiency in neutrophil apoptosis leads to delay or failure of inflammation resolution.^33, 34^ So we speculated that CHOP deficiency increased Ang II-induced cardiac inflammatory response, maybe partly by reducing neutrophil apoptosis and delaying of inflammation resolution.

CHOP-dependent cardiac injury may be mechanistically linked to NOX-induced oxidative stress. It is known that Ang II-mediated NAD(P)H oxidase activation can lead to generation of reactive oxygen species (ROS) and this has been widely implicated in vascular inflammation and fibrosis.^35^ There are also accumulating evidences showing that ROS can induce ER stress and CHOP expression. For example, ROS mediated CHOP-mediated ER stress underlie human lung adenocarcinoma cells apoptosis induced by resveratrol and arsenic trioxide.^36^ Cigarette smoke inducted human bronchial epithelial cell apoptosis via ROS-dependent ER Stress and CHOP.^37^ NADPH oxidase-dependent production of ROS induced ER stress in neutrophil-like HL60 cells.^38^

The study by Fu *et al.*^39^ reported that CHOP deficiency decreased cardiac hypertrophy, fibrosis and cardiac dysfunction compared with WT mice at 4 weeks after transverse aortic constriction (TAC). They also found that CHOP-deficient mice had less apoptotic cell death-related signaling compared with WT mice after TAC. Our results demonstrated that in Ang II-induced cardiac injury, CHOP deficiency increased cardiac inflammation, decreased neutrophil apoptosis at day 1 and increased fibrosis at day 7 compared with WT mice. The explanation of the difference may be the use of different models. TAC in the mouse is a commonly used chronic model for pressure overload-induced cardiac hypertrophy and heart failure.^40^ Along with the development of cardiac hypertrophy, a progressive increase in cardiomyocyte apoptosis was detected from 4 weeks after TAC, and there was fewer cardiomyocyte apoptosis within 1 week.^41, 42^ The study by Kitakaze and co-workers^42^ found sustained pressure overload induces prolonged ER stress, which may contribute to cardiac myocyte apoptosis during progression from cardiac hypertrophy to failure. In contrast, we and other showed that acute Ang II infusion caused an elevation of blood pressure along with increased inflammation and reparative fibrosis. Indeed, Ang II infusion triggered a set of inflammatory responses at day 1 after Ang II infusion and cardiac fibrosis at day 7 after Ang II infusion.^9, 20, 23^ Within 7 days of Ang II infusion, there is elevated neutrophil apoptosis but not cardiomyocyte apoptosis (Figure 6). In our study, we found ER stress in the early stage protected Ang II-induced acute cardiac injury. Taken together, different stimuli, that is, prolong *versus* acute caused irreversible or reversible ER stress leading to different outcomes of apoptosis. Prolong ER stress leads to structural cell (such as cardiomyocyte) apoptosis and remodeling, while ER stress in acute injury regulates apoptosis of short life inflammatory cell apoptosis and inflammation. Thus, our present study identified a novel role of ER stress in regulating inflammation resolution.

Consistent with our study, the effect of ER stress and CHOP in other diseases was also tissue- and cell type specific. CHOP-deficient mice were reported to be resistant to lung inflammation and injury induced by LPS infusion,^12^ whereas CHOP deficiency resulted in elevated LPS-induced inflammation and kidney injury.^14^ CHOP deficiency prevented unilateral ureteral obstruction-induced renal inflammation and fibrosis,^43^ while inactivation of CHOP promoted obesity-associated inflammation.^13^ Smooth muscle cells-CHOP-deficient mice displayed reduced proliferation in atherosclerosis.^44^ However, CHOP-null mutation increased proliferation and reduced apoptosis within the islets of *Leprdb/db* mice.^45^ It was recently reported that the ER stress pathway is also involved in the inflammatory response. CHOP, as a member of the C/EBP family of transcription factors, could directly regulate the expression of cytokines. For example, CHOP is a negative regulator of LPS-induced IL-6 expression in B cells.^46^ And CHOP is crucial for dendritic cell IL-23 expression.^47^ Thus, further studies exploring the molecular basis of ER stress and CHOP in protecting Ang II-induced cardiac inflammation and injury are required.

## Materials and Methods

### Animals and treatments

CHOP knockout (CHOP KO) mice on a C57B/L6 background were obtained from the Jackson Laboratory (Bar Harbor, ME, USA), and littermates were used as controls. Mice were bred and kept under specific pathogen-free conditions in the animal facility of the Beijing Institute of Heart, Lung and Blood Vessel Diseases. The Guide for the Care and Use of Laboratory Animals (National Institutes of Health Publication No. 85-23, 2006) was followed, and the study was approved by the Animal Care and Use Committee of Capital Medical University.

### Animal model and treatments

To induce hypertension, osmotic minipumps (Alzet Model 1007D; DURECT, Cupertino, CA, USA) filled with Ang II or acetic acid saline were placed subcutaneously to deliver Ang II at a concentration of 1500 ng/kg /min as described previously.^9, 22^ Systolic blood pressure was measured at the indicated day using a computerized mouse tail cuff system (BP98A; Softron, Tokyo, Japan) as described elsewhere.^21, 23^ Cardiac echocardiography was performed using the Vevo 2100 high-resolution microimaging system (VisualSonic, Toronto, ON, Canada) as described previously.^2^

### Generation of BM chimeric mice

BM transplantation was performed as described elsewhere.^48, 49^ Briefly, C57BL/6 WT mice and CHOP KO mice were killed with carbon dioxide narcosis. BM cells were collected from the femurs and tibias of donor mice by needle flushing and resuspended in RPMI-1640. Four hours after irradiation, recipient mice were intravenously injected with 1 × 10^7^ BM cells. The mice were then kept in a specific pathogen-free environment for another 8 weeks to reconstitute their BM with sterilized water and food. Four groups of chimeric mice were generated: BM^wild type (WT)^ to WT, BM^KO^ to WT, BM^WT^ to KO and BM^KO^ to KO.

### Flow cytometry and FACS sorting

Flow cytometry was performed using single-cell suspension, which was prepared as described with minor modifications.^50, 51, 52^ Briefly, mouse hearts were minced and digested with 1.6 mg/ml collagenase IA (Sigma Aldrich, Tokyo, Japan) and 200 *μ*g/ml DNase I (Roche, Indianapolis, IN, USA) in PBS at 37 °C for 50 min. Then cell suspensions were filtered and collected at 300 × *g* for 10 min. Mouse spleens were removed, triturated in PBS at 4 °C with the end of a 3-ml syringe and filtered through nylon mesh (BD Biosciences, San Jose, CA, USA). The cell suspension was centrifuged at 300 × *g* for 10 min at 4 °C. Red blood cells were lysed with ACK lysis buffer, and the splenocytes were washed with PBS. Flow cytometry was carried out using the following antibodies: PE anti-mouse F4/80 (Biolegend, San Diego, CA, USA), PE-cy7 anti-mouse Ly6G, PerCP-Cy5.5 anti-mouse CD45.2, PE-CF594 anti-mouse CD3e, Alexa 488 anti-mouse CD11b, APC-cy7 anti-mouse CD11b, V450 anti-mouse Ly6C, Alexa 488 anti-mouse CD31 and PE anti-mouse PDGF*α* (all from BD Biosciences). Flow cytometry data were acquired using BD LSRFortessa (BD Biosciences) and analyzed by BD FACSDiva software (BD Biosciences). FACS sorting was performed on a BD FACS Aria II System (BD Biosciences).

### TUNEL labeling

Apoptotic cells were identified by the DeadEnd Fluorometric TUNEL System (Promega, Madison, WI, USA) on frozen heart sections according to the manufacturer's protocol.^53^ Co-staining of TUNEL and anti-Gr1 (1 : 100), anti-F4/80 (1 : 100) (all from Abcam, Cambridge, MA, USA) and anti-*α*-actinin (1 : 800; Sigma Aldrich, St Louis, MO, USA) was performed to further clarify the cell type of apoptotic cells. Images were captured by a Leica TSC-SP5 laser-scanning confocal microscope (Leica, Wetzlar, Germany).

### Histology and immunohistochemical analysis

Mouse heart tissue was fixed in 4% paraformaldehyde, embedded in paraffin and sectioned. Heart sections (4 *μ*m) were stained with Masson's trichrome and picrosirius red reagents.^4^ Heart sections were stained with primary antibodies for *α*-SMA (1 : 200), TGF-*β* (1 : 200) (both from Santa Cruz Biotechnology, Santa Cruz, CA, USA) at 4 °C overnight, then with secondary antibodies at room temperature for 0.5–1 h and detected with 3,3′-diaminobenzidine.

### RNA extraction, whole-transcriptome sequencing and quantitative real-time PCR

Total RNA was extracted with TRizol (Invitrogen, Carlsbad, CA, USA) according to the manufacturer's protocol, followed by DNase I treatment to eliminate DNA contamination. Equal quantities of RNA from five samples in one group were mixed. Every group was detected one time. The mRNA was enriched and fragmented into short fragments (approximately 200 bp). The double-stranded cDNA was synthesized and purified before sequencing adaptors were ligated to the fragments. For quality control, an Agilent 2100 Bioanalyzer and ABI StepOnePlus Real-Time PCR System were used to qualify and quantify the sample library. The library products were finally sequenced via Illumina HiSeq 2000. No less than 11 M clean reads were obtained from each sample.

For quantitative teal-time PCR, 2 *μ*g of total RNA were reverse transcribed into cDNA using the GoScript reverse transcription system (Promega). The gene expression levels were analyzed by quantitative reverse transcriptase PCR (qRT-PCR) performed with 2 × SYBR Master Mix (Takara, Otsu, Shiga, Japan) using an iCycler iQ5 (Bio-Rad, Hercules, CA, USA). The primers used in this study are detailed in Table 1.

### Western blotting

Protein was extracted from cells using lysis buffer containing protease/phosphatase inhibitors. The protein lysates were separated by SDS-PAGE, transferred onto a PVDF membrane (Millipore, Billerica, MA, USA), and then blocked with 5% skim milk (BD Biosciences) in TBST for 1 h at room temperature. The membranes were incubated with indicated primary antibodies, including CHOP (1 : 1000), ATF4 (1 : 1000), Grp78/Bip (1 : 1000), BCL-XL (1 : 1000), BCL-2 (1 : 1000) (all from Cell Signaling Technology, Beverly, MA, USA) and GAPDH (1 : 1000; Santa Cruz) at 4 °C overnight, followed by incubation with IRDye-conjugated secondary antibodies (1 : 1000; Rockland Immunochemicals, Gilbertsville, PA, USA). Images were quantified using the Odyssey infrared imaging system (LI-COR Biosciences, Lincoln, NE, USA).

### Neutrophil isolation and assessment of apoptosis

Murine BM-derived neutrophils were sorted by flow cytometry.^54^ Briefly, BM cells were flushed from tibias and fibulas, filtered and suspended in RPMI-1640 1 × supplemented with 10% FBS. The cells were incubated with an antibody mix (488 anti-mouse CD11b, PE-cy7 anti-mouse Ly6G) for the selection of neutrophils for 15 min on ice and washed. Stained cells were analyzed and sorted by MoFlo with Summit 5.2 software (Beckman Coulter, Miami, FL, USA). Debris and dead cells were excluded by forward scatter, side scatter and PI (BD Biosciences) gating. Cell purity (CD11b^+^Ly6G^+^) was >95%, as assessed by flow cytometry.

Purified cells were resuspended in RPMI with 10% FBS and 50 U/ml streptomycin and penicillin. Cells were cultured in the presence of Ang II (1 *μ*mol/l) for indicated times. To assess the apoptosis of neutrophil, FACS was performed using BD LSRFortessa (BD Biosciences). Neutrophil apoptosis was calculated as the percentage of annexin V^+^ cells (both annexin V^+^PI^−^ and annexin V^+^PI^+^) to total cells at each time point.

### Statistical analysis

Data were expressed as the means±standard error of the mean (S.E.M.). Differences between groups were tested for statistical significance using Student's *t*-tests or one-way analysis of variance (ANOVA) followed by Newman–Keuls multiple comparison tests using GraphPad Prism 5.0. *P*<0.05 denoted the statistically significant difference.

## Figures and Tables

**Table 1:** Sequences of primers used in real-time PCR

**Gene**	**Forward**	**Reverse**
*Grp78/Bip*	5′-TTCCGCTCTACCATGAAACC-3′	5′-TCTTTTGTCAGGGGTCGTTC-3′
*ATF4*	5′-ATGGCCGGCTATGGATGATG-3′	5′-TCTGGCATGGTTTCCAGGTC-3′
*CHOP*	5′-GGAACCTGAGGAGAGAGTGTTC-3′	5′-AAGGTGAAAGGCAGGGACTC-3′
*GAPDH*	5′-GGTTGTCTCCTGCGACTTCA-3′	5′-GGTGGTCCAGGGTTTCTTACTC-3′
*Collagen I*	5′-CATGTTCAGCTTTGTGGACCT-3′	5′-GCAGCTGACTTCAGGGATGT-3′
*Collagen III*	5′-TCCCCTGGAATCTGTGAATC-3′	5′-TGAGTCGAATTGGGGAGAAT-3′
*Fibronectin*	5′-CGGAGAGAGTGCCCCTACTA-3′	5′-CGATATTGGTGAATCGCAGA-3′
*S100a8*	5′-GGAGTTCCTTGCGATGGTGA-3′	5′-TCCTTGTGGCTGTCTTTGTGAG-3′
*S100a9*	5′-AGATGGCCAACAAAGCACCT-3′	5′-TAAAGGTTGCCAACTGTGCT-3′
*CCL2*	5′-CCACTCACCTGCTGCTACTCAT-3′	5′-CTTCTTTGGGACACCTGCTGCT-3′
*CXCL1*	5′-ACCCAAACCGAAGTCATAGCC-3′	5′-TTGTCAGAAGCCAGCGTTCA-3′
*GRP94*	5′-GTCAAAAGAAAACGTTCGAAATCA-3′	5′-CCGCCGCAACATGTCTCT-3′
*GADD34*	5′-CGCCGCGTCAGGGTATAA-3′	5′-TGACTCAATCTGCGCCAACA-3′
*Xbp1*	5′-ACCCCGCCCGAGTTGA-3′	5′-GCGGGTATATTCATCACTTATTGGT-3′
*TNF-α*	5′-CACAAGATGCTGGGACAGTGA-3′	5′-TCCTTGATGGTGGTGCATGA-3′
*IL-6*	5′-TTCCATCCAGTTGCCTTCTTG-3′	5′-TTGGGAGTGGTATCCTCTGTGA-3′
*IL-10*	5′-CCAGGGAGATCCTTTGATGA-3′	5′-CATTCCCAGAGGAATTGCAT-3′
*IL-4*	5′-GGAGATGGATGTGCCAAACG-3′	5′-CGAGCTCACTCTCTGTGGTGTT-3′

## Abbreviations

ER stress: endoplasmic reticulum stress
Ang II: angiotensin II
CHOP: C/EBP homologous protein
BM: bone marrow
SMA: smooth muscle actin
TGF-*β*1: transforming growth factor-*β*1
WT: wild type
ATF6: activating transcription factor 6
GRP78: the 78-kDa glucose-regulated protein
CXCL: chemokine (C-X-C motif) ligand
CCL: chemokine (C-C motif) ligand
TAC: transverse aortic constriction
LPS: lipopolysaccharide
