# Supplementary material for: Endoplasmic reticulum stress in bone marrow-derived cells prevents acute cardiac inflammation and injury in response to angiotensin II
Source: Cell Death Dis. 2016 Jun 9;7(6):e2258–. doi: 10.1038/cddis.2016.164 (PMC5143392; doi:10.1038/cddis.2016.164)
Supplement: Supplementary Material [file cddis2016164x1.doc]

**Supplemental Figure legends**

**Supplemental FigureⅠ**Hypertension induced ER stress after Ang II infusion. **(A)** GRP98, GADD34 and Xbp-1 mRNA in Ang II-infused WT mouse hearts at day 1 determined by qRT-PCR (n=4 in each group). **(B)** Bip, ATF4 and CHOP mRNA levels expression were quantiﬁed at the indicated times after Ang II infusion by real-time PCR (n = 4 in each group). **P*<0.05 compared with Sham group.

**Supplemental Figure Ⅱ.** CHOP deficiency did not affect infiltration of inflammatory cells in saline-infused hearts. **(A)** Flow cytometry analysis of CD45^+^ leucocytes, CD45^+^CD11b^+^Ly6G^+^F4/80^-^neutrophils, CD45^+^CD11b^+^F4/80^+^macrophages and CD45^+^CD3^+^ T cells were performed in saline-infused WT and CHOP KO hearts at day 1 (n = 4 in each group). **(B)** Bar graph shows the percentage of cells in the heart (n = 4 in each group). NS indicates not significant, compared with WT group.

**Supplemental Figure Ⅲ** CHOP deficiency did not affect the inflammatory response in spleens. **(A)** Flow cytometry analysis of CD45^+^CD11b^+^Ly6C^+^ monocytes, CD45^+^CD11b^+^F4/80^+^ macrophages, and CD45^+^CD11b^+^Ly6G^+^F4/80^-^ neutrophils in Ang II-infused WT and CHOP KO spleens at day 1 (n = 4 in each group). **(B)** Bar graph shows the percentage of cells in the spleen (n = 4 in each group). **P*<0.05 compared with Sham group, NS indicates not significant, compared with WT group.

**Supplemental Figure Ⅳ.** CHOP deficiency did not affect blood pressure, cardiac hypertrophy or cardiac function in Ang II-treated mice. **(A**) Systolic blood pressure was measured by the tail-cuff method at different time points after Ang II infusion. **(B)** The ratios of heart weight to tibia length in WT and CHOP KO mouse at day 7 in sham or Ang II infusion group. **(C)** EF (ejection fraction) value (%) and FS(shortening fraction) value (%) of WT and CHOP KO mouse were evaluated at day 7 in sham or Ang II infusion group by cardiac ultrasonography. n=5 per group , **P*<0.05 compared with Sham group, NS indicates not significant.
