# Supplementary figures and images for: Endoplasmic reticulum stress in bone marrow-derived cells prevents acute cardiac inflammation and injury in response to angiotensin II
Source: Cell Death Dis. 2016 Jun 9;7(6):e2258–. doi: 10.1038/cddis.2016.164 (PMC5143392; doi:10.1038/cddis.2016.164)

**A**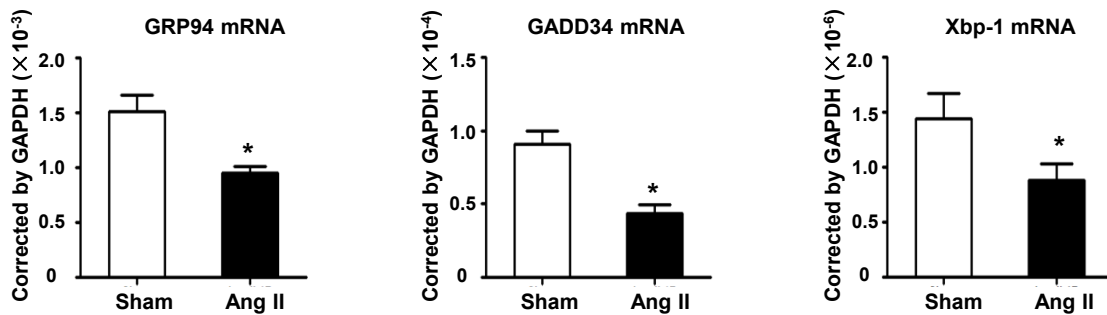**B**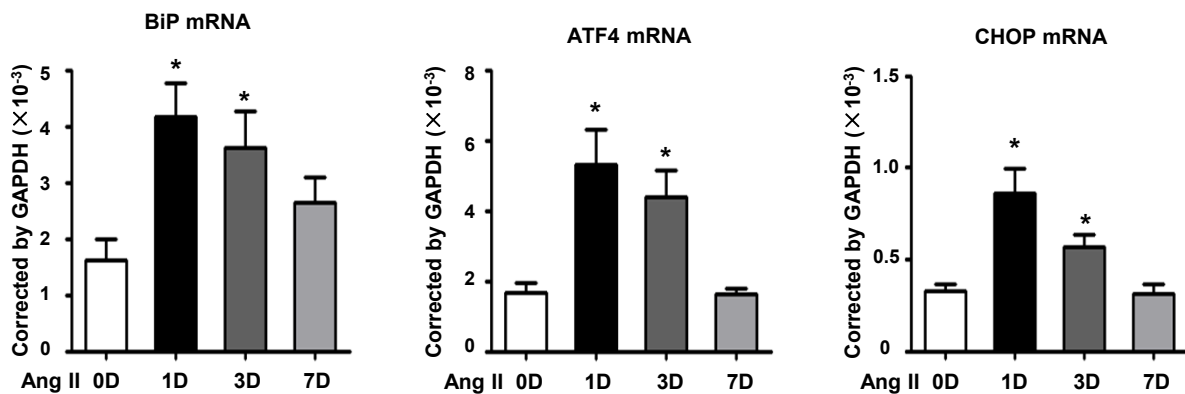

Supplement: Supplementary Figure 1 [file cddis2016164x2.pdf]

**A****WT Sham**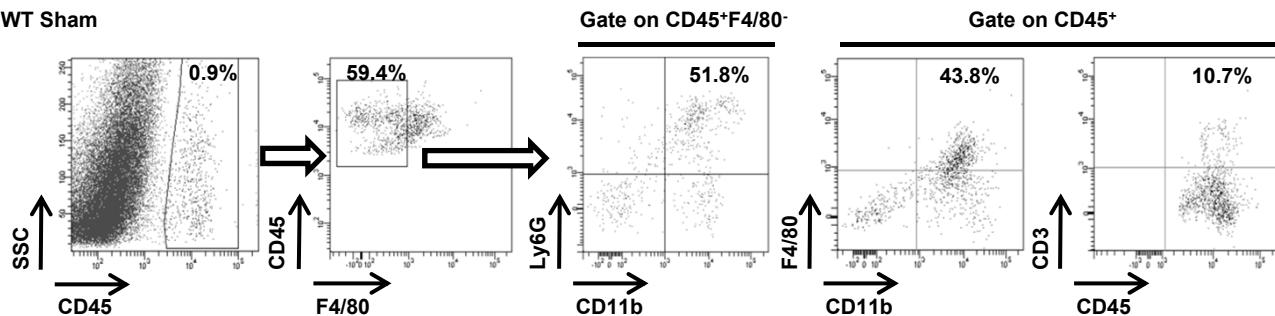**KO Sham**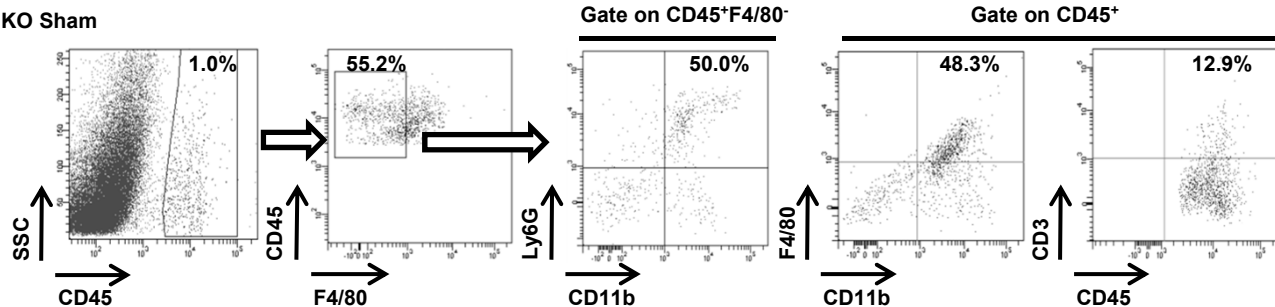**B**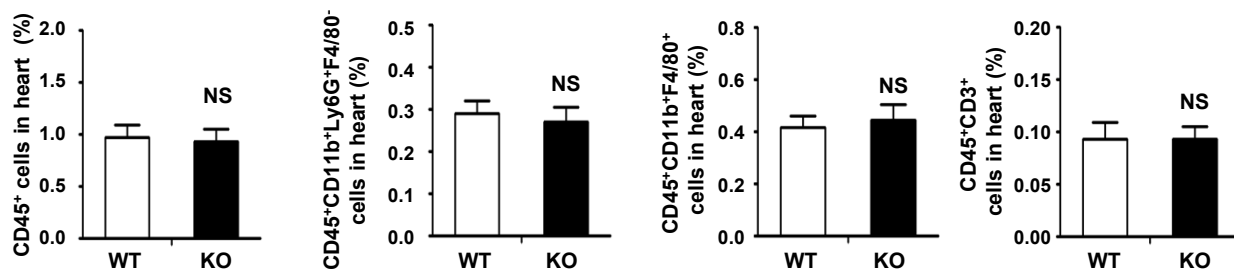

Supplement: Supplementary Figure 2 [file cddis2016164x3.pdf]

**A**Gate on CD45<sup>+</sup>Gate on CD45<sup>+</sup>F4/80<sup>+</sup>

WT Sham

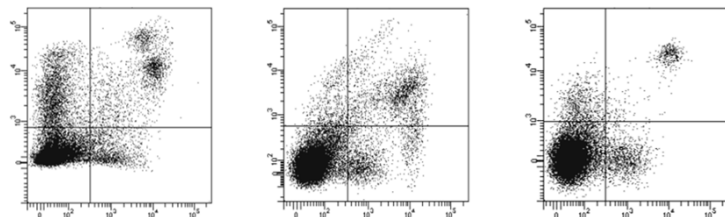

KO Sham

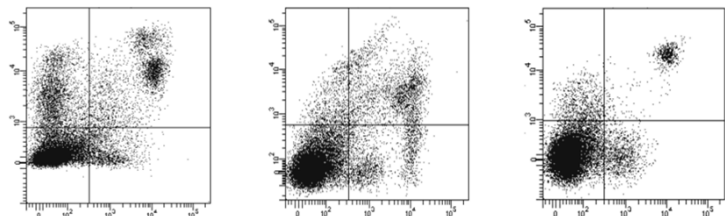

WT Ang II 1D

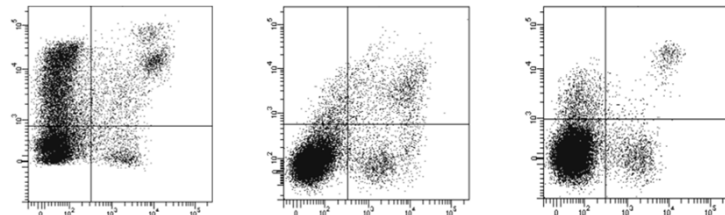

KO Ang II 1D

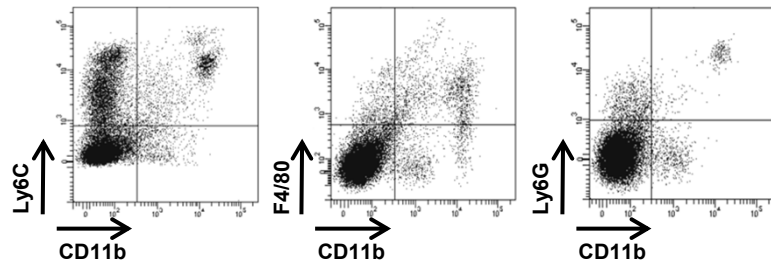**B**□ WT  
■ KO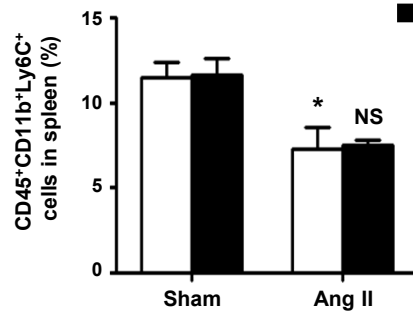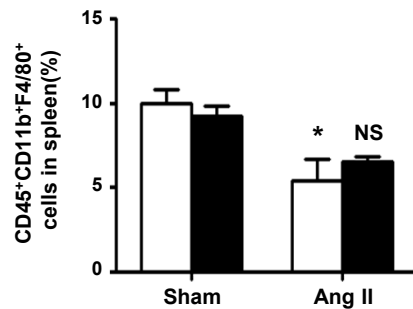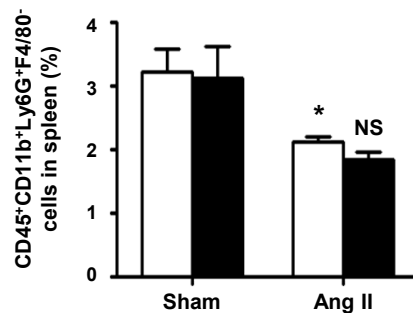

Supplement: Supplementary Figure 3 [file cddis2016164x4.pdf]

**A**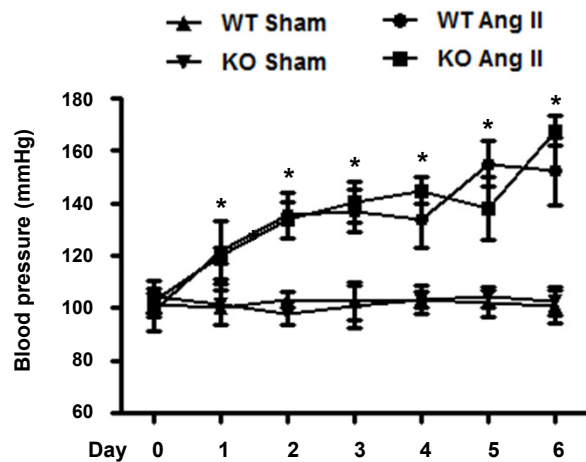**B**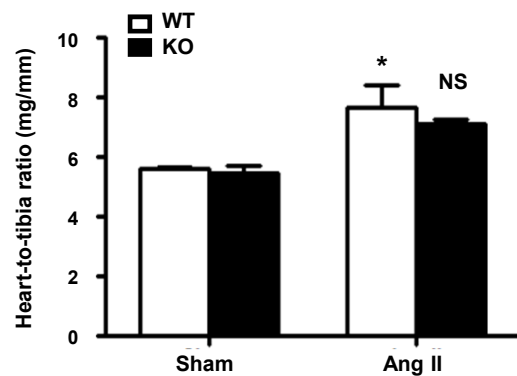**C**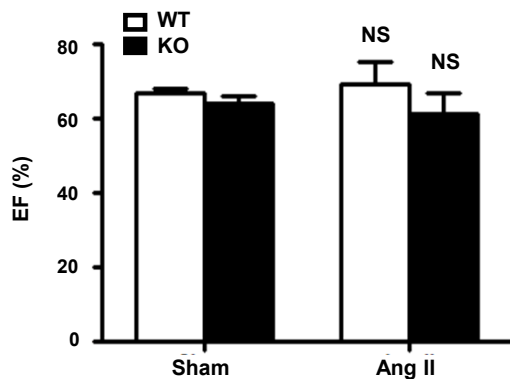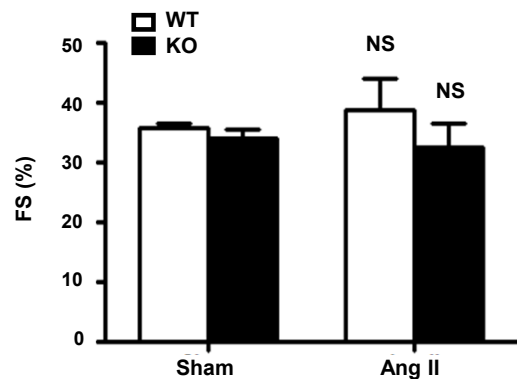

Supplement: Supplementary Figure 4 [file cddis2016164x5.pdf]
